# AliC and AliD of nonencapsulated *Streptococcus pneumoniae* enhance virulence in a *Galleria mellonella* model of infection by contributing to reactive oxygen species resistance

**DOI:** 10.3389/fcimb.2025.1583375

**Published:** 2025-06-11

**Authors:** Courtney D. Thompson, Md Fahim Khan, Lucas R. G. Crosby, Shelby G. Holcomb, Ana G. Jop Vidal, Jorge E. Vidal, Larry S. McDaniel, Lance E. Keller

**Affiliations:** ^1^ Department of Biological Sciences, Mississippi College, Clinton, MS, United States; ^2^ Department of Cell and Molecular Biology, University of Mississippi Medical Center, Jackson, MS, United States; ^3^ Center for Immunology and Microbial Research, University of Mississippi Medical Center, Jackson, MS, United States

**Keywords:** *Streptococcus pneumoniae*, oligopeptide transporter, *Galleria mellonella*, virulence factor, host-pathogen

## Abstract

**Introduction:**

Nonencapsulated *Streptococcus pneumoniae* (NESp) are isolated worldwide. Due to the lack of capsule in NESp strains the current vaccines, that target the pneumococcal capsule are ineffective. Some NESp contain the oligopeptide transporters AliC and AliD which are required for virulence through unknown mechanisms. AliC and AliD have been previously shown to reduce rates of phagocytosis and alter the transcriptome and proteome of MNZ41. We hypothesize that oligopeptide regulated genes are responsible for reduced phagocytosis and increased survival through resistance to reactive oxygen species (ROS).

**Methods:**

To test this a mutant library of AliC and AliD regulated genes was used in *in vitro* and *in vivo* models. ROS resistance was tested through quantifying bacterial counts after exposure to hydrogen peroxide (H_2_O_2_). A modified surface killing assay was also used to calculate resistance to phagocytosis of our mutant library. A *Galleria mellonella* larvae model of infection was used to determine survival curve analyses.

**Results:**

Two mutant genes in our library, ∆*lytFN1* (CDT04) and ∆*mgtC* (CDT05), displayed greater sensitivity to H_2_O_2_ killing and phagocytosis compared to wildtype MNZ41. Deletion of AliD in an AliD-expressing encapsulated strain reduced virulence.

**Conclusion:**

This research demonstrates that proteins encoded by genes regulated by AliC and AliD alter susceptibility to host-derived mechanisms for bacterial clearance and increases bacterial survival in response to ROS.

## Introduction

*Streptococcus pneumoniae*, commonly referred to as the pneumococcus, are Gram-positive bacteria that asymptomatically colonize the human nasopharynx (Keller et al., 2016). Although pneumococci are normally commensal organisms, pneumococci can disseminate into other tissues after colonization and cause a wide range of diseases such as otitis media (OM), pneumonia, and meningitis (Henriques-Normark and Tuomanen, 2013; Keller et al., 2016). The development and widespread use of pneumococcal vaccines that target the capsular polysaccharide have led to a decrease in invasive pneumococcal disease (IPD), but as a result, increased isolation of nonencapsulated *Streptococcus pneumoniae* (NESp) has been observed (Hicks et al., 2007; Song et al., 2013; Bradshaw and McDaniel, 2019). NESp lacks a capsule and can account for nearly 20% of all pneumococcal carriage isolates (Keller et al., 2016). NESp typically cause noninvasive infections but can cause IPD (Park et al., 2012; Park et al., 2014; Keller et al., 2016). NESp are categorized into two groups based on the gene content found in the capsular polysaccharide synthesis (*cps*) locus, an area between the conserved *dexB* and *aliA* genes (Park et al., 2014). Group I NESp have *cps* genes but do not produce polysaccharide capsules due to mutations in the *cps* locus (Park et al., 2014). Group II NESp do not have *cps* genes and encode the novel genes *pspK*, *aliC*, or *aliD* in their place (Park et al., 2012). Based on the inclusion or exclusion of these genes in the *cps* locus, Group II NESp are further divided into three groups known as null capsule clades (NCCs) (Park et al., 2012; Varghese et al., 2017). NESp are frequently isolated from the nasopharynx of asymptomatic carriers and are generally nonvirulent (Park et al., 2012; Park et al., 2014; Keller et al., 2016). However, in a U.S. study of IPD isolates, 0.61% were found to be nontypeable, and out of these NESp isolates, 82% were comprised of null capsule clade 2 (NCC2) strains (Park et al., 2014; Mohale et al., 2016). NCC2 strains contain the novel genes *aliC* and *aliD*, which encode for Ami-like surface proteins AliC and AliD that bind and deliver small oligopeptides to transmembrane permeases that import oligopeptides into the cell (Hathaway et al., 2004; Park et al., 2012; Hathaway et al., 2014; Mohale et al., 2016; Bradshaw et al., 2018; Alcorlo et al., 2024). Interestingly, *aliD* is also found in the *cps* locus of encapsulated strains of serotypes 25A, 25F, and 38, as well as in other streptococcal species (Hathaway et al., 2004; Bentley et al., 2006; Hilty et al., 2014).

AliC and AliD are part of the Ami permease system, which is an ATP-binding cassette (ABC) transporter encoded by the *amiACDEF* operon (Alloing et al., 1994). It is comprised of transmembrane proteins AmiC and AmiD, cytosolic ATPases AmiE and AmiF, and cell-membrane anchored lipoprotein AmiA that binds and concentrates oligopeptides, delivering them to AmiC and AmiD for import (Alloing et al., 1994; Claverys et al., 2000; Kerr et al., 2004). AliC and AliD expressed by NESp are paralogs of AmiA, and thus, the “Ali” nomenclature is derived from their “AmiA-like” homology (Hathaway et al., 2004; Kohler et al., 2016; Schmidt et al., 2019). Previous research has demonstrated that oligopeptide transporters in encapsulated pneumococcal strains contribute to an increase in virulence during meningitis infections (Schmidt et al., 2019). It has also been experimentally shown in animal models that NESp strains containing *aliC* and *aliD* have increased colonization rates and enhanced virulence during otitis media and pulmonary infections compared to their isogenic mutants (Bradshaw et al., 2018). Likewise, the presence of *aliC* and *aliD* have been shown to promote pneumococcal survival during exposure to chinchilla whole blood and human polymorphonuclear leukocytes (PMNs), to reduce complement C3b deposition on the bacterial surface, and to evade the immune system, permitting NESp to persist during IPD (Bradshaw et al., 2018; Thompson et al., 2023).

The ability to evade the host innate immune response and survive host mediated clearance is required to cause IPD. Experiments commonly use mouse models to test this but alternative *in vivo* models have been developed, such as zebrafish and invertebrate models. These alternative models contain innate immune responses and are good for high throughput virulence screens due to reduced cost and requirements compared to more traditional animal models (Evans and Rozen, 2012; Saralahti et al., 2014; Tsai et al., 2016). The clearance of the pneumococcus, both encapsulated and nonencapsulated, from the host is through phagocytosis and intracellular killing by macrophages and neutrophils (Barbuti et al., 2010). These phagocytes mediate killing by releasing defensins and lysosomes and by producing reactive oxygen species (ROS). ROS, such as superoxide anions, hydrogen peroxide (H_2_O_2_), and hydroxyl radicals participate in the oxidative burst response and aid in bacterial clearance (Marriott et al., 2008; Yesilkaya et al., 2013; Nguyen et al., 2017; Brooks and Mias, 2018). Although the mechanisms behind immune evasion by NESp are not well understood, the AliC and AliD proteins play an important role in this process, possibly through both reducing phagocytosis and resistance to intracellular clearance (Bradshaw et al., 2018; Thompson et al., 2023). We have previously shown that regulation of *cbpAC* by pneumococci expressing AliD decreases phagocytosis through reducing deposition of C-reactive protein (Thompson et al., 2023). Based on available transcriptomics and proteomics data, AliC and AliD alter the expression of numerous proteins with diverse functions, such as proteins used in metabolism, virulence, and some proteins of unknown function (Bradshaw et al., 2018; Nasher et al., 2018; Thompson et al., 2023). We hypothesize that other AliC and AliD regulated genes aid in virulence through reducing phagocytosis and increasing resistance to ROS upon phagocytosis.

Widespread vaccine use has caused increases in pneumococcal disease caused by non-vaccine type from roughly 6% to 20% (Mrabt and Guedes, 2025). While longitudinal studies that identify NCC subtypes are lacking, there are studies that include NESp in their analysis before or after vaccine introduction. These studies indicate an increase of NESp from 0.61% to 2% after vaccine introduction (Park et al., 2014; Kawaguchiya et al., 2024). This increase in prevalence makes the study of how NESp causes disease and persist within the host necessary. Our study focuses on determining which AliC and AliD regulated genes are utilized in virulence and resistance to reactive oxygen species upon phagocytosis. Here we use a *Galleria mellonella* (greater wax moth) larvae model of infection, which allows for screening of potential virulence factors that aid in avoiding the innate immune response. We demonstrate that two AliC and AliD regulated genes, *lytFN1* and *mgtC*, are required for full virulence and reduction in phagocytosis. Furthermore, our data indicate resistance to ROS is mediated by *lytFN1*. Altogether, this study demonstrates that AliC and AliD regulated genes alter susceptibility to host-derived mechanisms for bacterial clearance, providing further insight into how NESp can cause IPD.

## Results

### Gene selection

**Figure 1:**
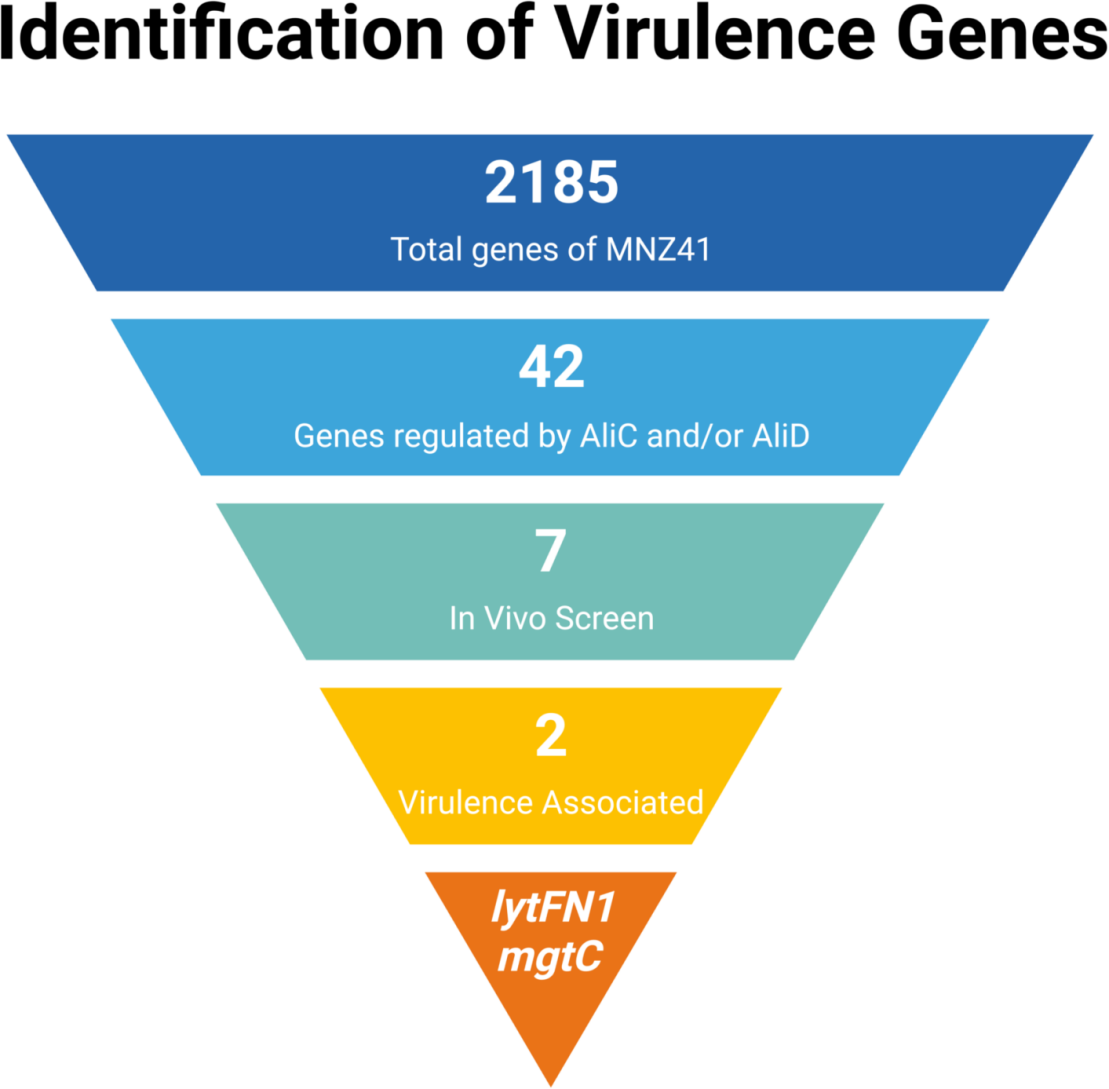
Gene selection and identification of virulence genes. This figure displays a flowchart demonstrating the selection process that led to the identification of virulence genes *lytFN1* and *mgtC*. The 2,185 genes of MNZ41 were screened using proteomics and transcriptomics. Analysis of the ‘omics data determined that 42 of those genes were regulated by oligopeptide binding protein AliD. Out of the 42 AliD-regulated genes, 7 mutants were created and used in *in vivo* screening, which determined that the 2 genes *lytFN1* and *mgtC* were important in virulence. Figure created using BioRender (https://biorender.com/).

Published proteomics and transcriptomics data of NESp strain MNZ41 or 110.58 (Accession CP007593), which express AliC and AliD, were used to identify genes regulated by AliC and AliD (Bradshaw et al., 2018; Nasher et al., 2018). Of the 2,185 coding regions of NESp strain MNZ41, it was determined that at least 42 genes were regulated by either AliC or AliD. Out of the 42 regulated genes, 7 differentially expressed genes of interest were chosen and mutants created to use in *in vivo* and *in vitro* experiments (**Figure 1**).

### Kaplan-Meier analysis of survival in the *Galleria mellonella* model infected with pneumococci

**Figure 2:**
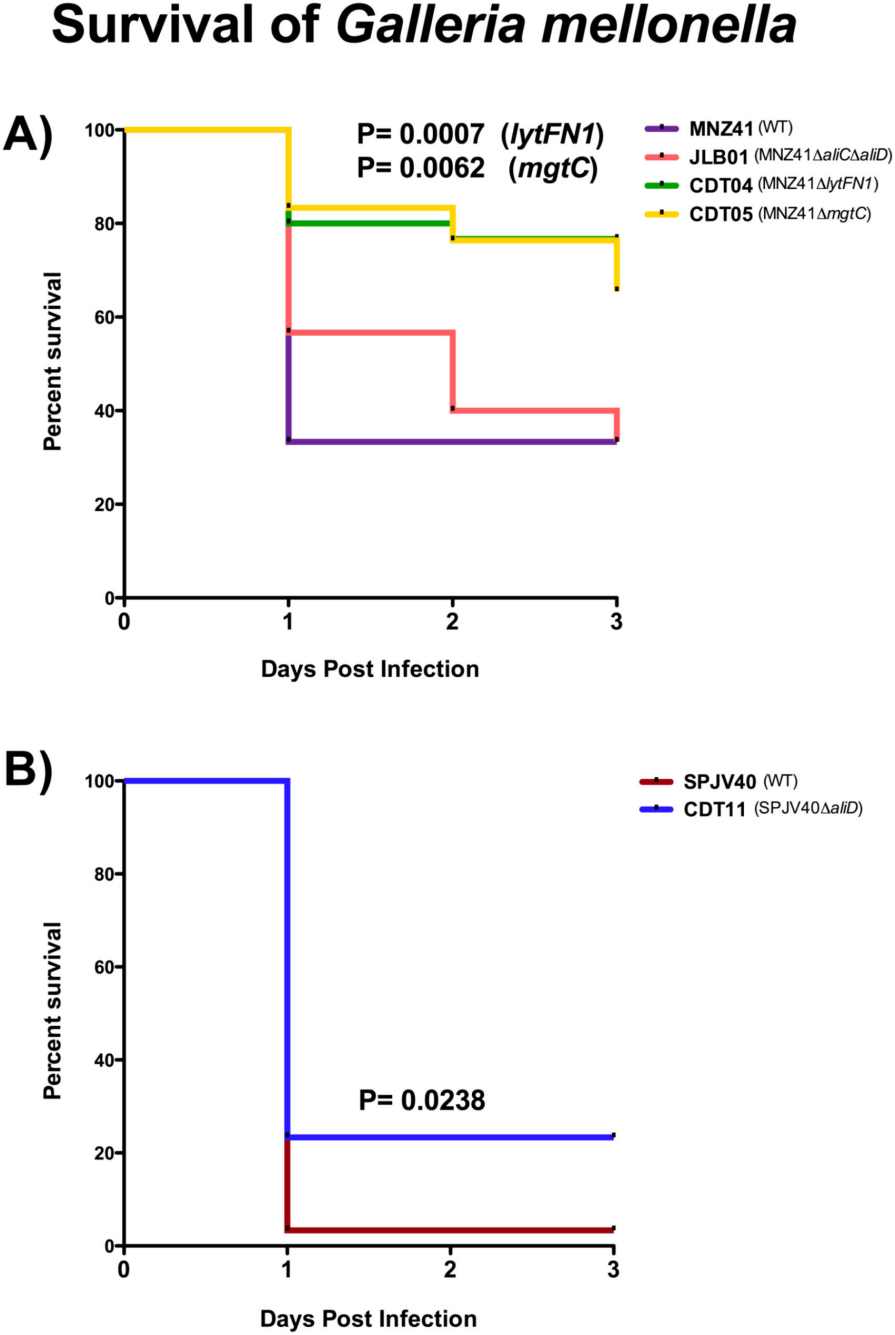
Kaplan-Meier analysis of survival in the *Galleria mellonella* model infected with wildtype pneumococci and respective mutants. *Galleria mellonella* larvae were infected with each pneumococcal strain, and survival was monitored for 72 hours. Kaplan-Meier survival curves were determined for WT NESp (MNZ41) and isogenic mutants JLB01 (MNZ41Δ*aliC*Δ*aliD*), CDT04 (MNZ41Δ*lytFN1*), and CDT05 (MNZ41Δ*mgtC*) at a concentration of 5×10^8^ CFU/mL **(A)**. Kaplan-Meier survival curves were also determined for WT encapsulated serotype 38 SPJV40 and its isogenic mutant CDT11 (SPJV40Δ*aliD*) at a concentration of 1.85×10^7^ CFU/mL **(B)**. Data from at least three biological replicates, n=10 larvae per replicate, were used to calculate median values for graphs. Survival curves were analyzed for statistical significance using the log-rank test. A *P* value of <0.05 was considered to be statistically significant.

To characterize the AliC and AliD regulated genes of interest, a high throughput *G. mellonella* larval model of infection was used as a mimic of the complete innate immune system (Cools et al., 2019). Kaplan Meier survival curve analysis was used to assess differences in survival between WT MNZ41 and all mutants, JLB01 and CDT01 through CDT07. No matter the dosage, WT MNZ41 mortality was highest within the first 24 hours and had a survival rate of 33.33% on day 3 (**Figure 2A**). In comparison, the larvae injected with the double *aliC/aliD* mutant JLB01 also had a survival rate of 33.33% on day 3, but mortality occurred less rapidly than WT MNZ41 (**Figure 2A**). There was no significant difference in mortality between larvae injected with MNZ41 and JLB01 (*P*= 0.7467). Analysis also indicated that there were high rates of survival in larvae injected with the *lytFN1* mutant CDT04 (76.67% survival) and the *mgtC* mutant CDT05 (66.67% survival) regardless of the concentration of bacteria injected (**Figure 2A**). Survival of larva infected with CDT04 and CDT05 was significantly higher compared to WT MNZ41 with *P* values of 0.0007 and 0.0062, respectively, indicating that genes *lytFN1* and *mgtC* play a role in virulence (**Figure 2A**). Of interesting note, CDT02 (MNZ41Δ*SP6UMMC_07241*), a hypothetical protein, became more lethal than WT MNZ41 upon gene deletion (3.33% survival, *P*= 0.0062), meriting further investigation in future studies (**Supplementary Figure 1**). Of the remaining CDT mutants tested, there was no significant difference in larval survival when compared to MNZ41 (**Supplementary Figure 1**).

To further examine the role of *aliD* in encapsulated strains that naturally express *aliD*, these same analyses were performed using strains SPJV40 (serotype 38) and CDT11 (SPVJ40Δ*aliD*) (Thompson et al., 2023). Kaplan Meier survival curve analysis determined that larvae challenged with the WT serotype 38 strain SPJV40 had high levels of mortality by day 1 and had a survival rate of 3.33% (**Figure 2B**). In comparison, CDT11 (SPJV40Δ*aliD*) had a significantly higher survival rate of 23.33% (*P*= 0.0238) (**Figure 2B**). Additionally, a growth curve analysis was performed to determine if there was any deficit in growth. As determined by area under the curve analysis we found that there was no significant growth defect when comparing mutant growth to wildtype MNZ41. However, there was a significant increase in growth when comparing JLB01 (MNZ41Δ*aliC*Δ*aliD*) to WT MNZ41 (**Supplementary Figures 2A, D**). There was also no growth defect when *aliD* was deleted from encapsulated strain SPJV40 or when *aliD* was expressed in R36A (CDT08) (**Supplementary Figures 2B, C**).

### Murine model of colonization and infection

**Figure 3:**
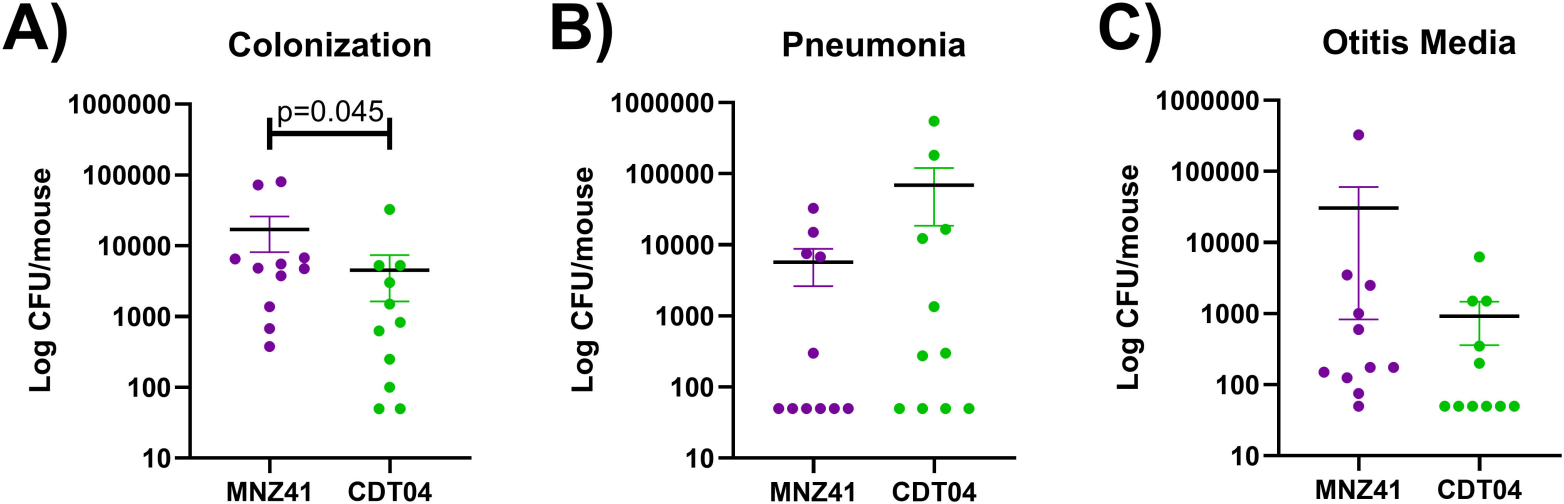
Murine colonization and infection with wildtype and *lytFN1* mutant. Mice were infected intranasally with either wildtype MNZ41 or the isogenic *lytFN1* mutant (CDT04) in an aspiration pneumonia model. After 48 hours, mice were euthanized and tissues collected for bacterial enumeration. Significantly fewer bacteria were collected from the nasopharynx when infected with CDT04 compared to MNZ41 **(A)**. Infection with either MNZ41 or CDT04 demonstrated no differences in the number of pneumococci collected from either the lungs **(B)** or the middle ear **(C)**. Data was collected from two independent infections. Error bars represent the standard errors of the means.

To determine if the *lytFN1* gene was also required for colonization or disease in a mammalian system, a pneumonia mouse model was used. Bacterial burdens of the nasopharynx, lung, and middle ear were determined after infection with WT MNZ41 or the *lytFN1* mutant CDT04. Colonization was significantly reduced when the *lytFN1* gene was deleted as fewer bacteria were recovered from the nasopharynx at 48 hpi (**Figure 3A**). Despite reduced colonization efficiency of the *lytFN1* mutant there was no significant difference in the number of bacteria recovered from either the lungs or middle ear of infected mice (**Figures 3B, C**).

### Pneumococcal gene regulation

**Figure 4:**
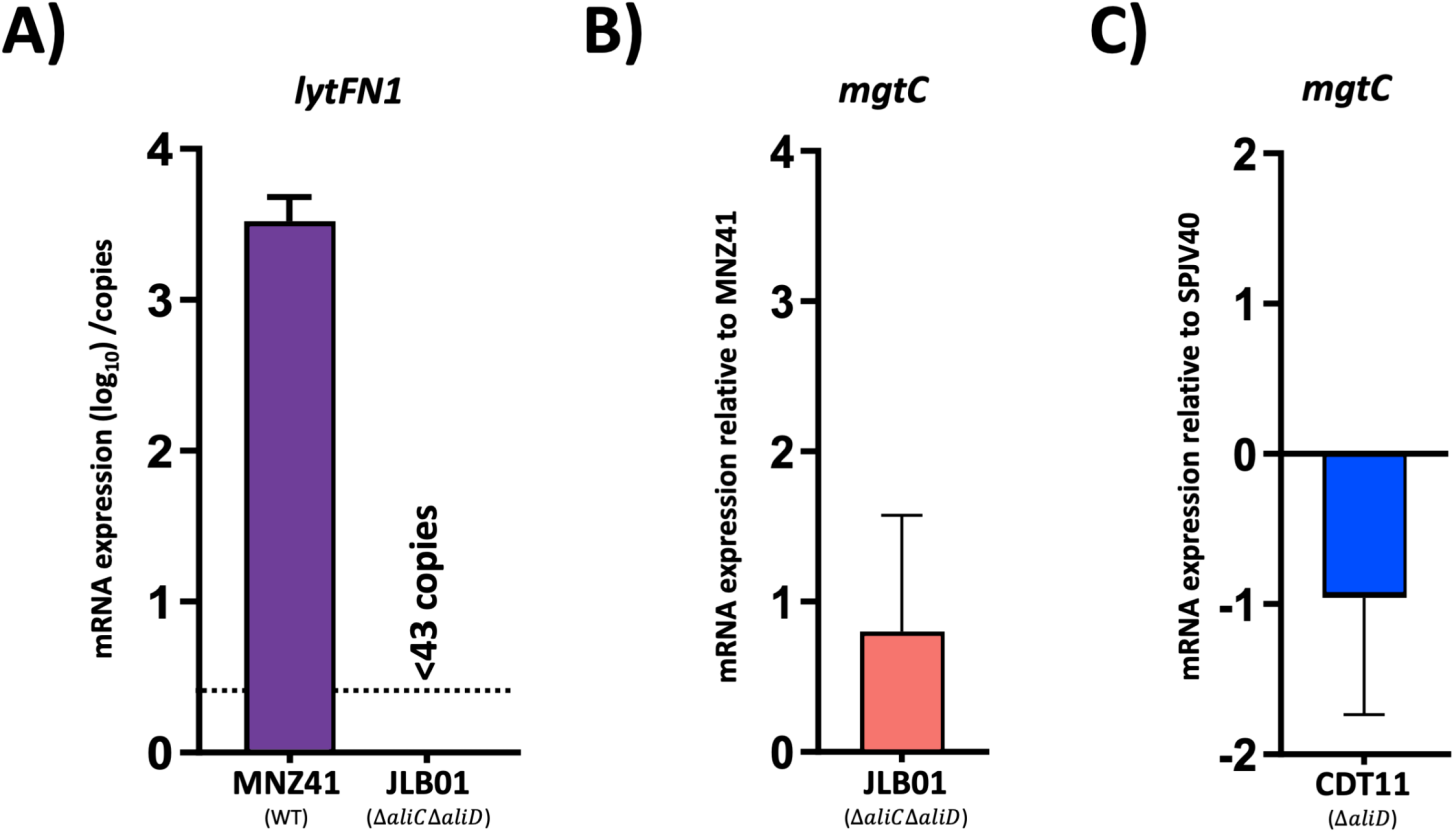
Pneumococci gene regulation. AliC/AliD regulation of *lytFN1* and *mgtC* in NESp and AliD regulation of *mgtC* in an encapsulated serotype 38 strain were analyzed by RT-qPCR. Total RNA was extracted from MNZ41 (NESp) and JLB01 (MNZ41Δ*aliC*Δ*aliD*) **(A, B)** and from SPJV40 (serotype 38) and CDT11 (SPJV40Δ*aliD*) **(C)**. cDNA was generated and utilized as a template in RT-qPCRs with primers that amplified the *lytFN1* and *mgtC* genes, respectively. Total number of *lytFN1* transcripts were normalized to the *gyrA* transcript numbers and presented as total number of transcripts when above limit of detection. Average C_T_ values were normalized to the value for the *gyrA* gene. The fold differences were calculated using the comparative C_T_ (2^-ΔΔC^_T_) method. Panels show data from two independent biological replicates. Error bars represent the standard errors of the means.

Selection of genes for mutagenesis in our mutant library was performed using available transcriptomics and proteomics data. To assess AliC and AliD regulated genes *lytFN1* and *mgtC*, which we have shown to be required for lethal infections, RT-qPCR was performed. The *lytFN1* gene is present in approximately 7% of pneumococcal strains and is present in MNZ41 but not SPJV40 (Croucher et al., 2015). In the absence of *aliC* and *aliD*, we were unable to detect any transcripts of *lytFN1* in MNZ41. Therefore, we were unable to determine fold change for *lytFN1* expression. As indicated in **Figure 4A**, the total number of *lytFN1* transcripts for MNZ41 was over 3000 while *lytFN1* transcripts in JLB01 was below the limit of detection. Interestingly, we were unable to detect a significant difference in expression of *mgtC* in either MNZ41 or SPJV40 when *aliC* and/or *aliD* were deleted (**Figures 4B, C**). Both *aliC* (SP6UMMC_05342) and *aliD* (SP6UMMC_05352) are located in the *cps* locus and influence the expression of *lytFN1* (SP6UMMC_10841) but not *mgtC* (SP6UMMC_08188) in tested conditions.

### Phagocytosis

**Figure 5:**
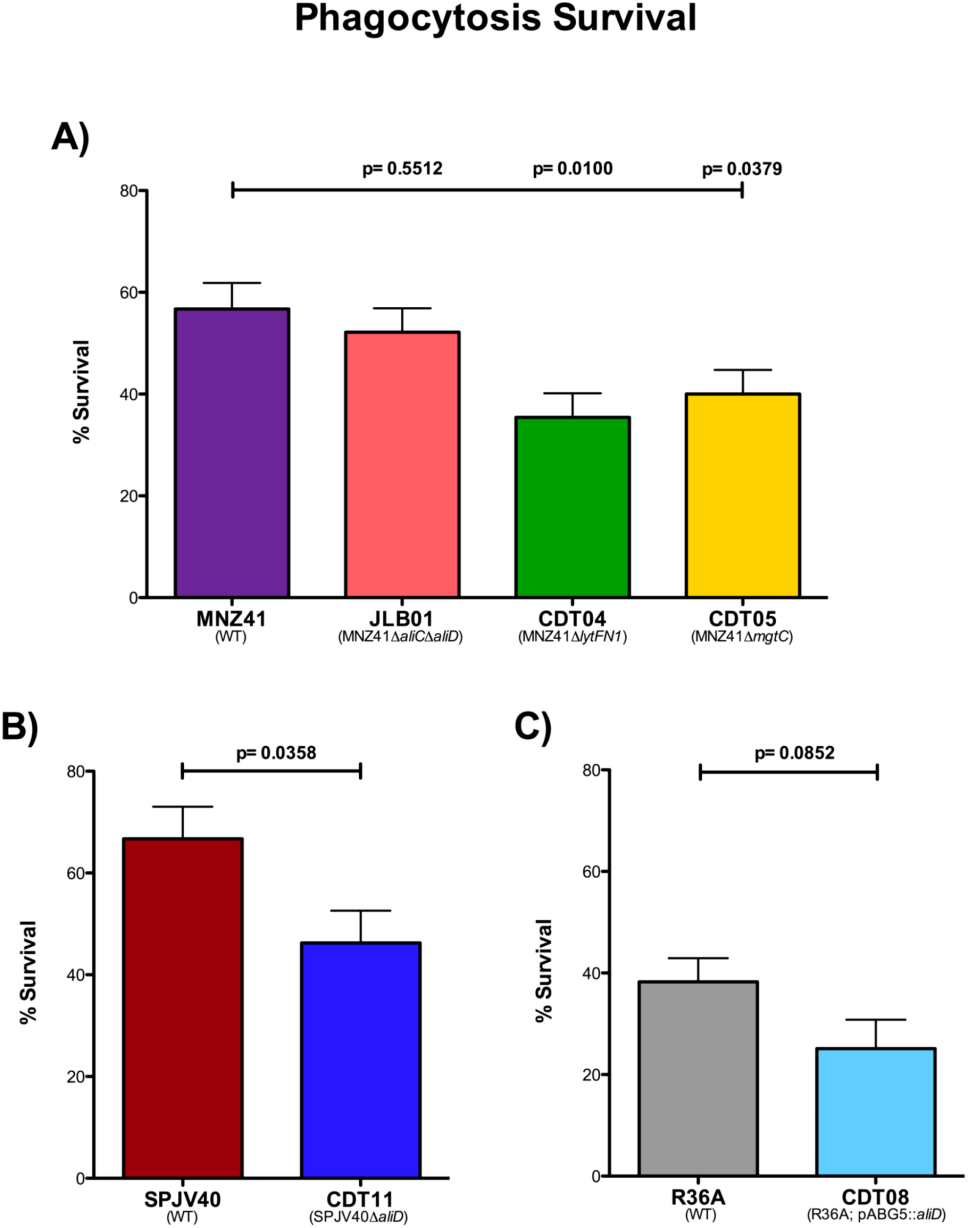
Modified surface killing assay. Pneumococcal strains were exposed to differentiated HL-60 cells at a ratio of 1:100, and phagocytosis survival percentages were calculated. The percentages of survival after phagocytosis of strains MNZ41 (WT NESp), JLB01 (MNZ41Δ*aliC*Δ*aliD*), CDT04 (MNZ41Δ*lytFN1*), and CDT05 (MNZ41Δ*mgtC*) were calculated by comparing the CFU of strains incubated with and those incubated without neutrophils **(A)**. The percentages of survival after phagocytosis of strains SPJV40 (serotype 38) and CDT11 (SPJV40Δ*aliD*) were calculated by comparing the CFU of strains incubated with and those incubated without neutrophils **(B)**. The percentages of survival after phagocytosis of strains R36A (unencapsulated laboratory strain) and CDT08 (R36A;pABG5::*aliD*) were calculated by comparing the CFU of strains incubated with and those incubated without neutrophils **(C)**. Bar graphs are representative of results from three independent experiments performed in triplicate. Error bars represent the standard errors of the means.

We have demonstrated that the genes *lytFN1* and *mgtC* are important for virulence in our larvae model. Therefore, we wanted to examine the ability of *lytFN1* and *mgtC* to resist phagocytosis by neutrophils. The percent survival was determined in the WT MNZ41, JLB01 (MNZ41Δ*aliC*Δ*aliD*), CDT04 (MNZ41Δ*lytFN1*), and CDT05 (MNZ41Δ*mgtC*) after exposure to differentiated HL-60 cells, a neutrophil-like cell line (Chaplinski and Niedel, 1982). In NESp strain MNZ41, the deletion of *aliC/aliD* did not cause a significant decrease in survival after phagocytosis (52.16%) compared to WT MNZ41 (56.73%) (**Figure 5A**). However, after the deletion of genes *lytFN1* or *mgtC*, there were significant decreases in survival after exposure to HL-60 cells (35.42%, *P*=0.0100 and 40.01%, *P*=0.0379, respectively) in comparison to WT MNZ41 (**Figure 5A**). The single *aliC* and *aliD* mutants, JLB02 (MNZ41Δ*aliC*) and JLB04 (MNZ41Δ*aliD*), along with the remaining CDT mutants were also tested for survival after phagocytosis but did not have significant decreases in survival in comparison to WT MNZ41 (**Supplementary Figure 3**).

Using the same experiment, we examined survival of encapsulated serotype 38 strain SPJV40 and its *aliD* mutant after phagocytosis. Upon exposure to HL-60 cells, serotype 38 WT strain SPJV40 had a survival rate of 66.73% (**Figure 5B**). In comparison, the isogenic *aliD* deletion mutant (CDT11) had a significantly decreased survival rate (46.27%, *P*=0.0358) (**Figure 5B**). We further wanted to recapitulate these results in a nonencapsulated strain that does not naturally express *aliD*, so an *aliD* expression vector was introduced into R36A (CDT08). Surprisingly, there was no significant difference in survival of R36A (38.25%) compared to 25.10% survival for CDT08 (R36A; pABG5::*aliD*) (**Figure 5C**).

### H_2_O_2_ resistance

**Figure 6:**
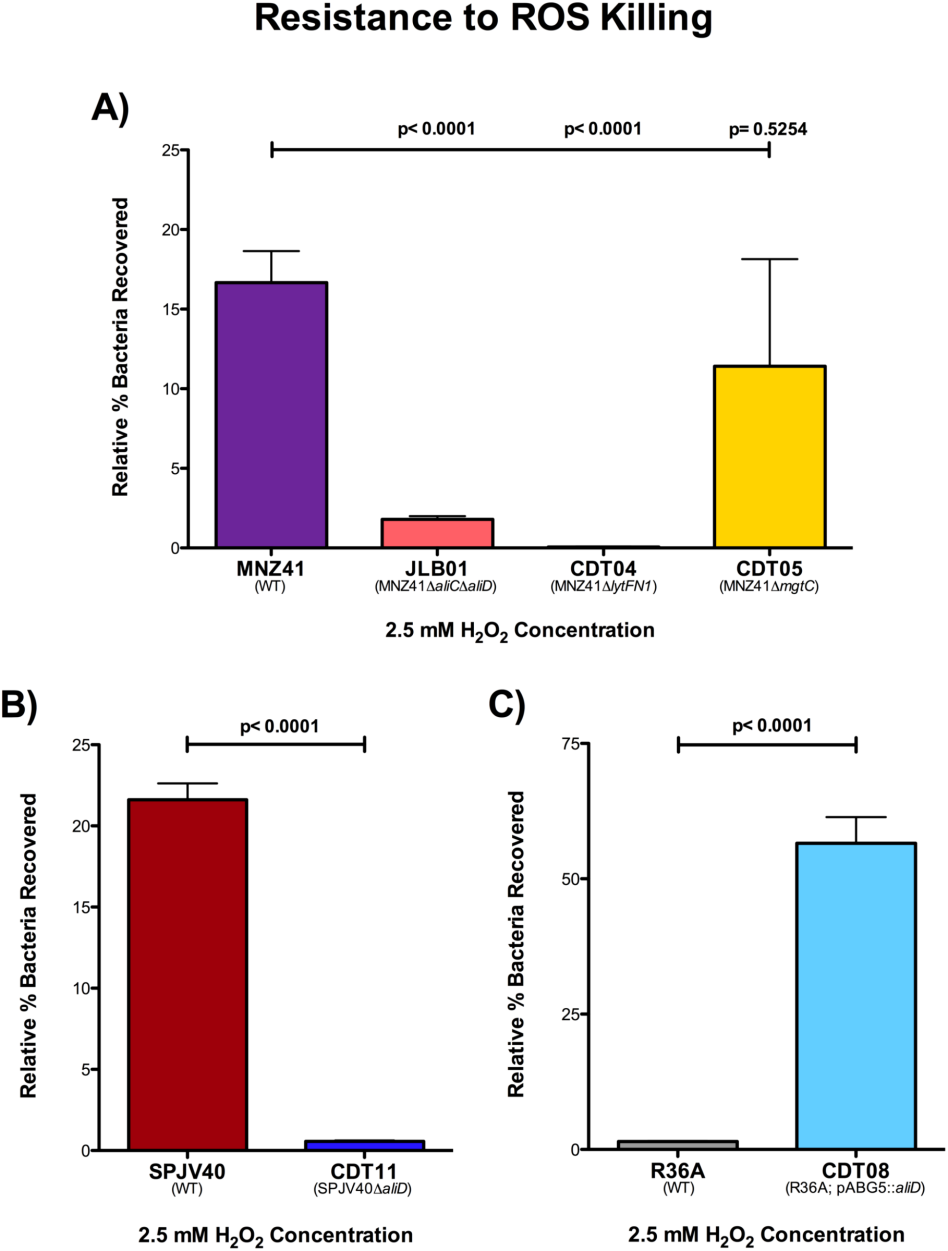
H_2_O_2_ resistance. Pneumococcal strains were treated with 2.5 mM of H_2_O_2_ in THY or with THY only for 2 hours and serial dilutions were plated on BA. Resistance to H_2_O_2_ was calculated for NESp strains MNZ41 (WT), JLB01 (MNZ41 Δ*aliC*Δ*aliD*), CDT04 (MNZ41Δ*lytFN1*), and CDT05 (MNZ41Δ*mgtC*) by comparing the CFU of strains incubated with and those without H_2_O_2_ **(A)**. Resistance to H_2_O_2_ was calculated for serotype 38 strains SPJV40 (WT) and CDT11 (SPJV40Δ*aliD*) by comparing the CFU of strains incubated with and those without H_2_O_2_ **(B)**. Resistance to H_2_O_2_ was calculated for unencapsulated laboratory strains R36A (WT) and CDT08 (R36A; pABG5::*aliD*) by comparing the CFU of strains incubated with and those without H_2_O_2_ **(C)**. Experiments were performed in triplicate, and the data are shown as the mean of triplicate wells. A *P* value of <0.05 was considered to be statistically significant.

We have determined that *lytFN1* and *mgtC* aid in virulence by avoiding neutrophil-mediated clearance. Upon phagocytosis, one of the main mechanisms by which neutrophils kill bacteria is through increased production of ROS, such as H_2_O_2_. Therefore, we next wanted to determine survival of our WT MNZ41 and isogenic mutants after exposure to H_2_O_2_. We grew our samples in 2.5 mM H_2_O_2_ for two hours and then enumerated on BA. We observed that our WT NESp MNZ41 were recovered at 16.67%, but in comparison, our *aliC/aliD* double mutant (JLB01) and our *lytFN1* mutant (CDT04) had significantly decreased survival rates of 1.80% and 0.061%, respectively (*P* < 0.0001 for both mutants) (**Figure 6A**). The *mgtC* mutant (CDT05) was recovered at 11.41% and was statistically nonsignificant compared to the WT MNZ41 (**Figure 6A**). We also tested the other JLB and CDT mutants to determine their resistance to ROS killing, and we saw that JLB02 (MNZ41Δ*aliC*) and JLB04 (MNZ41Δ*aliD*) both had significantly reduced survival compared to WT MNZ41 with survival rates of 0.026% and 5.04% (*P* < 0.0001 and *P*=0.0008) (**Supplementary Figure 4**).

We also examined resistance to ROS killing in the encapsulated serotype 38 strain SPJV40, CDT11 (SPJV40Δ*aliD*), unencapsulated laboratory strain R36A, and CDT08 (R36A;pABG5::*aliD*). Our results determined that SPJV40 had a survival rate of 21.61%, while its *aliD* mutant (CDT11) had a significant decrease in survival at 0.565% (*P* < 0.0001) (**Figure 6B**). When we examined ROS resistance in R36A, we saw that unencapsulated strain R36A had a survival rate of 1.21%. However, when we tested the strain in which we expressed *aliD* in R36A (CDT08), we saw a significant increase in survival (56.27%, *P* < 0.0001) (**Figure 6C**). These results further elucidate the role of AliD in virulence.

## Discussion

Here, we utilize the *Galleria mellonella* larvae model of infection, which is useful for high throughput *in vivo* screening, to identify AliC and AliD regulated genes required for virulence. Our results indicate that pneumococcal genes *lytFN1* and *mgtC* are required for virulence in a larvae model of infection. Furthermore, these genes aid in virulence by increasing survival when exposed to neutrophil-like cells and increase resistance to reactive oxygen species (ROS). Available ‘omics data were used to identify potential gene targets that had significantly varied gene expression when *aliD* was deleted or activated. Creation and testing of seven different isogenic mutants in a *G. mellonella* model allow for rapid identification of pneumococcal virulence genes. This study is the first to use the *G. mellonella* model to test virulence of nonencapsulated *S. pneumoniae* and identify *lytFN1* and *mgtC* as pneumococcal virulence factors. The *G. mellonella* model has previously been used to examine virulence in numerous bacteria, including encapsulated pneumococci (Peleg et al., 2009; Senior et al., 2011; Alghoribi et al., 2014; Cools et al., 2019; Leal et al., 2023). The presence of an innate immune system makes the model useful for identifying bacterial factors that promote systemic survival. Calculation of Kaplan Meier survival curves allows for a useful metric to determine changes in virulence. We found that regardless of the infectious dose of bacteria injected into the larvae, a lethal infection in over 50% of the larvae infected did not occur when either *lytFN1* or *mgtC* was deleted from MNZ41 (**Figure 2A**). This indicates that these two genes are essential to overcome the innate immune response in the larvae model.

To further verify that the larva model can be used to identify possible pneumococcal virulence factors, we further tested virulence in a mouse model. We chose to further examine the LytFN1 mutant as it is predicted to be a choline binding surface protein and would directly interact with the host. Use of an intranasal pneumonia model allows us to determine colonization efficiency and the development of both otitis media and pneumonia. There was a significant reduction in colonization efficiency of the *lytFN1* mutant CDT04 (4.5 × 10^3^ CFU/mouse) compared to the wildtype MNZ41 (1.7 × 10^4^ CFU/mouse). While there was reduced colonization of CDT04, there was no significant impact on the ability of the pneumococcus to cause either otitis media or pneumonia. NESp strains are typically associated with colonization or noninvasive pneumococcal disease and contain unique genes that aid in colonization (Keller et al., 2013). LytFN1 was required for efficient colonization in the mouse model. Additional research would be needed to determine if there are long term cost to bacterial fitness of the *lytFN1* mutant. The mechanism of *lytFN1* in ROS resistance also needs to be further examined as well as assess whether lytFN1 expression in more pathogenic pneumococci influences their virulence.

To examine if these genes aid in resisting the human innate immune system, we used the promyeoloblast cell line HL-60 in a modified surface killing assay. This cell line was chosen because it can be differentiated into neutrophil-like cells. Neutrophils are responsible for migrating to the site of infection, identifying foreign cells, and engulfing the invading cells. These phagocytes mediate killing by releasing granules such as defensins and lysosomes, releasing ROS, and recruiting other immune cells (Marriott et al., 2008; Nguyen et al., 2017; Brooks and Mias, 2018). Upon phagocytosis, the enzyme NADPH oxidase in neutrophils consumes oxygen and generates toxic ROS that participate in the oxidative burst response, aiding in bacterial clearance (Yesilkaya et al., 2013; Nguyen et al., 2017). Utilizing a modified surface killing assay with these cells, we are able to demonstrate a significant reduction in survival of *lytFN1* and *mgtC* mutants compared to WT MNZ41 (**Figure 5A**) (Genschmer et al., 2019). This further shows that the *G. mellonella* model is useful for testing an innate immune response to pneumococcal infections.

Interestingly, we observed no difference in phagocytosis in the AliC and AliD double mutant JLB01 despite *lytFN1* expression in JLB01 being undetectable (**Figure 4A**). Deletion of *lytFN1* reduced survival in our phagocytosis model, therefore other genes regulated by AliC and AliD could increase phagocytosis survival despite the lack of *lytFN1*. We also observed no significant change in *mgtC* expression when AliC and AliD were deleted (**Figure 4**). Expression of *mgtC* through AliC and AliD independent pathways may explain why no significant differences in JLB01 mortality compared to MNZ41 are observed. Previous data concerning MNZ41 and JLB01 infections have utilized bacterial counts to determine differences in bacterial virulence. While JLB01 has significantly reduced survivability in numerous animal models compared to WT MNZ41, this study used mortality as an endpoint. Due to this, changes in Kaplan Meier curves were not noted in our AliC and AliD mutant JLB01. The bacterial burden may be reduced, but direct quantification in our larval model was not performed. This corresponds with systemic infection data in a mouse model. Rapid bacterial clearance of JLB01 but not MNZ41 was observed with no change in murine mortality based on Kaplan Meier analysis (Thompson et al., 2023). While JLB01 required slightly higher amounts for lethal infections compared to WT MNZ41, the difference was minimal. Lethal infection only occurred when large bacterial challenges were used, which overwhelmed the larval innate immune system. This indicates that the *G. mellonella* larvae model is useful in identifying genes or mutations that are required for lethality but may not be appropriate for examining carriage and clearance unless bacterial quantification is also performed. Therefore, deletion of the two identified genes do not result in lethal infections and are essential for mortality.

The gene *lytFN1* is present at about 7% frequency in both encapsulated and nonencapsulated pneumococci (Croucher et al., 2015). *lytFN1* encodes the caspase superfamily domain protein peptidase C14, and although not yet determined, the expected function of *lytFN1* is to degrade peptidoglycan and play a role in cell death (Nasher et al., 2019). Research into prokaryotic strains containing proteins with peptidase C14 caspase domains is limited and is mainly described in the cyanobacteria *Microcystis aeruginosa*. *M. aeruginosa* caspase homologs have been shown to cleave proteins involved in toxin-antitoxin systems, but are not described as virulence mechanisms requiring future research of *lytFN1* (Klemenčič et al., 2021). This caspase homolog may induce apoptotic pathways in eukaryotic cells following endocytosis, which can be further explored. Our other gene of interest, *mgtC*, is a known virulence factor in *Salmonella enterica* serovar Typhimurium (Snavely et al., 1991). Previous data have shown that the *mgtCB* operon is carried by the *Salmonella* SPI-3 pathogenicity island, and since MgtC-like proteins are found in numerous bacteria, *mgtC*-related genes have more than likely been acquired through horizontal gene transfer (Blanc-Potard and Groisman, 1997; Blanc-Potard and Lafay, 2003). In *Salmonella enterica* serovar Typhimurium, *mgtC* is a virulence factor that is required for growth in a medium with low magnesium levels and is essential for *Salmonella* intramacrophage survival (Snavely et al., 1991; Moncrief and Maguire, 1998). Data has also shown that *mgtC* modulates the activity of the host P-type ATPase, which has significant effects on cellular ion homeostasis and membrane potential (Günzel et al., 2006; Alix and Blanc-Potard, 2007). Intracellular survival is not thought to be required for *S. pneumoniae* pathogenesis, so *mgtC* may play another role in this system. Neutrophils limit magnesium availability to inhibit bacterial survival upon phagocytosis. Therefore, *mgtC* may increase *S. pneumoniae* magnesium acquisition which increases survival independent of ROS resistance (Maloney and Valvano, 2006; Blanc-Potard and Groisman, 2021). Our data also indicates that deletion of other AliC and AliD regulated genes—*hpf, msbA*, *malX*, and *ytrB*— had no changes in virulence with the exception of *SP6UMMC_07241* (**Supplementary Figure 1**). Deletion of this hypothetical gene induced a hypervirulent phenotype compared to the wildtype strain. Upon further analysis, we found that the gene sequence for *SP6UMMC_07241* has homology to carbamoylphosphate synthase, a gene used in the arginine synthesis pathway. Previous research has shown that when arginine is at high concentrations, AliB expression is repressed (Günzel et al., 2006). Deletion of *SP6UMMC_07241* may reduce available arginine, which leads to increased expression of oligopeptide transporters, resulting in a hypervirulent state. Examination of the role *SP6UMMC_07241* in virulence merits future investigation.

Upon phagocytosis, the main mechanism of clearance of the pneumococcus, both encapsulated and nonencapsulated, from the host is through intracellular killing by macrophages and neutrophils (Barbuti et al., 2010). The NADPH oxidase found in neutrophils and macrophages uses oxygen to generate toxic ROS such as superoxide anions, H_2_O_2_, and hydroxyl radicals that participate in the oxidative burst response (Forman and Torres, 2001; Forman and Torres, 2002; Yesilkaya et al., 2013; Okumura and Nizet, 2014; Nguyen et al., 2017). The AliC and AliD regulated gene of interest, *mgtC*, plays a role in intramacrophage survival in other bacteria. Therefore, we wanted to test ROS resistance in our pneumococcal strains by growing strains in 2.5 mM H_2_O_2_ for 2 hours and calculating survival. Superoxide anions and H_2_O_2_ damage proteins and iron-sulfur clusters of dehydratases, which release free iron that reacts with H_2_O_2_, producing hydroxyl radicals, the most reactive of all ROS that targets DNA (Yesilkaya et al., 2013; Okumura and Nizet, 2014; Nguyen et al., 2017). Neutrophils also contain a granule-localized enzyme, myeloperoxidase, that converts H_2_O_2_ into hypochlorous acid upon the respiratory burst, enhancing the clearance of the invading pathogen (Nguyen et al., 2017). Our studies of pneumococcal virulence show that when grown in 2.5 mM H_2_O_2_, strains that possess AliC and AliD are more resistant to killing by H_2_O_2_ (**Figure 6**). This does not directly translate to resistance to phagocytic killing since JLB01 (MNZ41::*ΔaliCΔaliD*) was more sensitive to H_2_O_2_ compared to wildtype, but there was no difference in survival between wildtype MNZ41 and JLB01 following exposure to the differentiated HL-60 cell line. It is possible that wildtype and JLB01 are phagocytosed at similar rates and that H_2_O_2_ independent methods of clearance are be utilized. Although the mechanisms behind immune evasion are not well understood, this ability to resist killing by some forms of ROS production allows pneumococci to persist within a host and gives a selective advantage over other pathogens.

**Figure 7:**
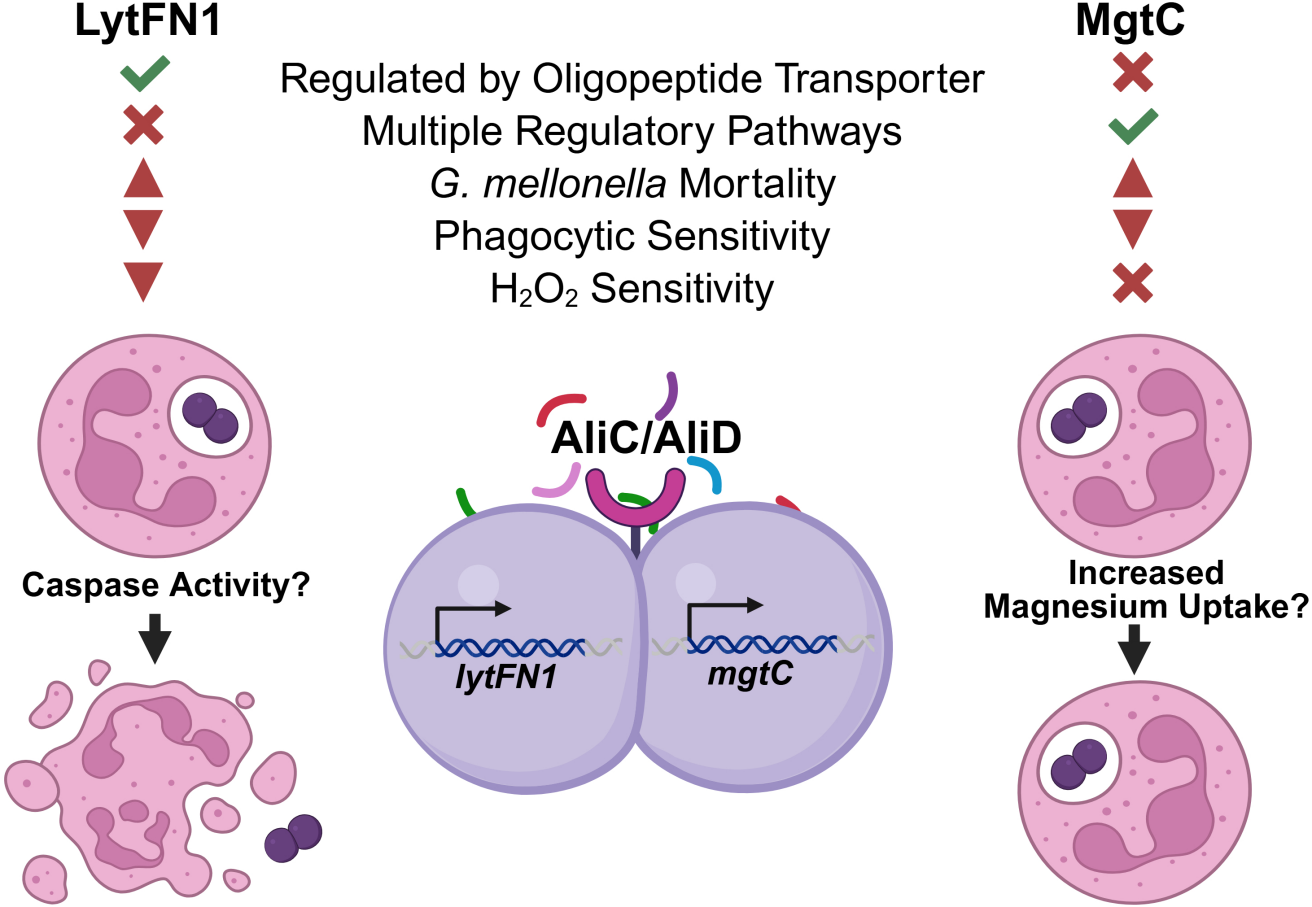
Model of mechanism. Genes *lytFN1* and *mgtC* were identified as potential virulence factors using the *Galleria mellonella* infection model. *In vitro* experiments identified deficiencies in survival following phagocytosis when either of these genes are deleted along with susceptibility to H_2_O_2_ in the *lytFN1* mutant. *S. pneumoniae* strains expressing *lytFN1*, which contains homology to peptidase C14 caspase, may activate apoptosis leading to neutrophil death and bacterial survival. The *mgtC* gene facilitates magnesium uptake, which can promote intracellular survival following phagocytosis independently of H_2_O_2_ mediated clearance mechanisms. Figure created using BioRender (https://biorender.com/).

Previous work has shown the importance of oligopeptide binding proteins AliC and AliD for virulence in different animal models and for the regulation of virulence genes (Bradshaw et al., 2018; Thompson et al., 2023). The available transcriptomics and proteomics data show numerous other genes regulated through AliC and AliD that may likewise contribute to the virulence of the strains that express AliC and AliD (Bradshaw et al., 2018; Nasher et al., 2018). In this study, we have demonstrated that AliC and AliD regulated genes *lytFN1* and *mgtC* aid in pneumococcal virulence through reducing neutrophil-mediated clearance. This is done in part by enhancing resistance to ROS, but apoptosis induction and enhanced magnesium import may also play a role (**Figure 7**). Through this research, we have gained a deeper insight into how NESp cause disease and how pneumococci are able to resist phagocytosis. Future studies will examine the hypervirulent hypothetical gene *SP6UMMC_07241* and other genes regulated by other oligopeptide transporters. AliC and AliD are diverse gene regulators that respond to environmental conditions, leading to unique gene expression profiles that promote survival in different conditions. Development of an oligopeptide transporter antagonist may provide novel treatment options that suppress immune avoidance mechanisms, allowing for efficient host derived clearance.

## Materials and methods

### Bacterial strains and growth conditions

Pneumococcal strains used in this study are described in **Table 1**. Strains were cultured in Todd Hewitt medium with 0.5% yeast extract (THY) or on blood agar (BA) and incubated at 37°C with 5% carbon dioxide. Strains grown to mid-log phase were collected in 1 mL aliquots containing 20% glycerol for storage at -80°C or were directly utilized in the assays.

**Table 1:** Description of pneumococcal strains used in this study .

Strain	Description	Antibiotic Resistance Marker^a^	Reference
MNZ41	NESp Carriage Isolate	Tmp (50 µg/ml)	(Bradshaw et al., 2018)
JLB01	MNZ41Δ*aliC* Δ*aliD*	Spec (300 µg/ml)	(Bradshaw et al., 2018)
JLB02	MNZ41Δ*aliC*	Kan (500 µg/ml)	(Bradshaw et al., 2018)
JLB04	MNZ41Δ*aliD*	Spec (300 µg/ml)	(Bradshaw et al., 2018)
CDT01	MNZ41Δ*hpf*; ribosome hibernation factor	Kan (500 µg/ml)	This study
CDT02	MNZ41Δ*SP6UMMC_07241*; hypothetical	Kan (500 µg/ml)	This study
CDT03	MNZ41Δ*msbA*; ABC transporter	Kan (500 µg/ml)	This study
CDT04	MNZ41Δ*lytFN*1; autolysin	Kan (500 µg/ml)	This study
CDT05	MNZ41Δ*mgtC*; Mg^2+^ transport ATPase	Kan (500 µg/ml)	This study
CDT06	MNZ41Δ*maIX*; carbohydrate metabolism	Kan (500 µg/ml)	This study
CDT07	MNZ41Δ*ytrB*; ATP binding	Kan (500 µg/ml)	This study
R36A	Unencapsulated derivative of D39	None	(Avery et al., 1944)
CDT08	R36A; pABG5::*aliD*	Kan (500 µg/ml)	(Thompson et al., 2023)
SPJV40	Serotype 38 pneumonia isolate	ND	(McCurdy et al., 2021)
CDT11	SPJV40 Δ*aliD*	Erm (1 µg/ml)	(Thompson et al., 2023)

*^a^* NESp, Nonencapsulated *Streptococcus pneumoniae*; Tmp, trimethoprim; Spec, spectinomycin; Kan, kanamycin; ND, not determined; Erm, erythromycin.

### Genetic manipulation

Specific mutagenesis details for the pneumococcal “JLB” strains have been described previously (Bradshaw et al., 2018). In summary, JLB01 (the MNZ41 AliC/AliD double mutant) was created by allelic replacement of *cps* with a spectinomycin resistance cassette. JLB02 (MNZ41 AliC mutant) and JLB04 (MNZ41 AliD mutant) were generated by allelic replacement of *aliC* or *aliD* with a kanamycin resistance cassette or spectinomycin resistance cassette, respectively. Origin details of pneumococcal strains SPJV40 and its AliD mutant have also been described (McCurdy et al., 2021; Thompson et al., 2023). Deletion of *aliD* from SPJV40 was created by allelic replacement with an erythromycin resistance cassette.

Creation of the AliC/AliD regulated gene knockouts (CDT mutants) was completed through Golden Gate cloning. Primers were designed with the open-source website Benchling to amplify DNA fragments for each gene knockout. Deletion of the *lytFN1* (CDT04) and *mgtC* (CDT05) genes from MNZ41 was performed by an in-frame allelic replacement with kanamycin resistance cassette. Primer pairs lytFN1_Up_F and lytFN1_Up_R were used to amplify the upstream homologous region of *lytFN1*, while primer pairs lytFN1_Down_F and lytFN1_Down_R were used to amplify the downstream region of *lytFN1* using chromosomal DNA of NESp MNZ41 as a template. Primer pairs mgtC_Up_F and mgtC_Up_R were used to amplify the upstream homologous region of *mgtC*, while primer pairs mgtC_Down_F and mgtC_Down_R were used to amplify the downstream region of *mgtC* using chromosomal DNA of NESp MNZ41 as a template. The kanamycin cassette was amplified from plasmid pPEPY using primer pairs Kan_BsaI_F and Kan_BsaI_R or Kan_BsmBI_F and Kan_BsmBI_R (Keller et al., 2019). Restriction enzyme BsaI or BsmBI sites were added into primer (underlined and bolded in sequence) for Golden Gate assembly of the three fragments. The assembled DNA fragments were then transformed into competent MNZ41. The primer pairs used for amplification of upstream and downstream fragments for the remaining CDT mutants (CDT01 (MNZ41Δ*hpf*), CDT02 (MNZ41Δ*SP6UMMC_07241*), CDT03 (MNZ41Δ*mbsbA*), CDT06 (MNZ41Δ*malX*), and CDT07 (MNZ41Δ*ytrB*)) are listed in **Supplementary Table 1**. Transformation of pneumococcal strains was performed in competence medium (THY, 0.2% BSA, 0.2% glucose, 0.2% CaCl_2_). Strains were grown to an OD_600_ of 0.1 and competence induced with 100 ng of competence stimulating peptide 1 (CSP1) and 100 ng of CSP2 before addition of the DNA construct (100 ng). Transformants were selected on BA supplemented with the appropriate antibiotic (**Table 1)**.

Using the *Escherichia coli*-pneumococcus shuttle expression vector pABG5, AliD was expressed in nonencapsulated laboratory strain R36A to produce CDT08. AliD was PCR amplified from MNZ41 genomic DNA with primers AliD_EcoRI_pABG5_F and AliD_PstI_pABG5_R and cloned into the pABG5 vector. Prior to ligation, the fragments were purified and digested with restriction enzymes EcoRI and PstI (underlined and bolded in primer sequences). The pABG5::*aliD* plasmid was transformed into *E.coli* and verified by sequencing. All primer sequences are listed in **Supplementary Table 1**.

### *In vivo* infection model

Healthy *Galleria mellonella* or “greater wax moth” larvae with no sign of disease were used for infections within two weeks of receipt. All wildtype and isogenic mutants were used to test virulence in a *G. mellonella* larvae model of infection. Each strain was tested at four different concentrations of bacteria suspended in 1X phosphate buffered saline (PBS) (5×10^8^ CFU/mL, 1.6×10^8^ CFU/mL, 5.5×10^7^ CFU/mL, and 1.85×10^7^ CFU/mL) to determine Kaplan Meier survival curves at various infectious doses. Healthy, consistently sized larvae were separated into random groups of ten, weighed, and placed into sterile Petri dishes (n=10 larvae per dose per strain per experiment). Larvae were placed at 4-8°C for five minutes before piercing the segment below the last set of prolegs with a 50 *μ*L Hamilton syringe. Each larva was injected with 10 *μ*L of bacteria. Control groups of ten larvae were each injected with 10 *μ*L of filter-sterilized 1X PBS. After injections, the larvae were incubated in the dark at 37°C, and larval mortality was monitored at 24, 48, and 72 hours post infection (hpi). Larvae were considered dead if they were melanized and/or unmoving and unable to reorient when placed on their backs. For each strain, data from at least three independent experiments were combined. Kaplan-Meier survival curves were generated and analyzed for statistical significance using the log-rank test.

Six- to eight-week-old C57BL/6J mice were obtained from Jackson Lab and allowed to acclimate for a minimum of 24 hours before infection. Mice were lightly anesthetized with isoflurane and intranasally challenged with 100 *μ*L of PBS containing 10^7^ CFU of MNZ41 or CDT04. At 48 hpi mice were euthanized and the nasopharynx, lungs and middle ears were collected. Samples were serially diluted and plated on BA containing 5 *μ*g/ml of gentamicin for bacterial enumeration. Two independent infection studies containing male and female mice were performed. No difference between male and female mice were observed, and data were combined for each condition for further analysis. All animal studies were performed in accordance with protocols approved by the University of Mississippi Medical Center Institutional Animal Care and Use Committee.

### RNA extraction, RT-qPCR analysis, and quantification of mRNA transcripts

Bacteria utilized in the reverse transcription quantitative PCR (RT-qPCR) studies were suspended in a 2-fold volume of RNAprotect bacterial reagent (Qiagen), centrifuged, and stored at -20°C. Total RNA was extracted using the RNeasy Plus minikit (Qiagen) according to the manufacturer’s protocol. DNA was removed using 1 U of DNase I (Promega) at 37°C for 30 minutes, followed by incubation with DNase stop solution (Promega) at 65°C for 10 minutes. RNA integrity was verified by gel electrophoresis and the concentration obtained by using a NanoDrop spectrophotometer (Thermo Scientific). cDNA was generated with iScript Reverse Transcription Supermix (Bio-Rad) and was used as a template for RT-qPCR reactions. RT-qPCR was performed with the PerfeCTa SYBR green SuperMix kit (Quanta Bio) and a CFX96 real-time PCR detection system (Bio-Rad). RT-qPCR analysis of the *lytFN1* expression levels of MNZ41and JLB01 (MNZ41Δ*aliC*Δ*aliD*) was performed using primers F_RT_lytFN1 (CGAAGGCGATGTGAACTATCT) and R_RT_lyFN1 (GTTCCAGCATGACAACAATCC). Analyses of the *mgtC* expression levels of MNZ41, SPJV40, and their mutant derivatives were performed using primers F_RT_mgtC (GGTTTAGAGAGAGGGAGCAAATC) and R_RT_mgtC (ACCTGATATAACTTGAGCTCCTAATC). Melting curves were generated by a cycle of 95°C for 3 minutes, followed by 40 cycles of 95°C for 15 seconds, 58°C for 30 seconds, and 72°C for 30 seconds. The relative mRNA expression level was normalized to the constitutive expression level of the *gyrA* gene (primers F_RT_gyrA [CCCATAGTTGCACGTCCTGT] and R_RT_gyrA [TCGTGGTGGTAAGGGAATGC]) and calculated by the comparative threshold cycle (*C_T_*) (2^-ΔΔCT^) method for two independent biological replicates performed in triplicate (Livak and Schmittgen, 2001).

To quantify the *lytFN1* mRNA transcript, cDNA was generated from 400 ng of RNA of each strain, performed as mentioned above. This cDNA was run along with a standard curve prepared with genomic DNA that was extracted from WT NESp strain MNZ41 using the QIAamp DNA Mini protocol (Qiagen). DNA standards utilized were as follows: 4.28×10^5^, 4.28×10^4^, 4.28×10^3^, 4.28×10^2^, 4.28×10^1^, and 4.28 genomic equivalent (GEq) per reaction using a genome size of 2,130,446 base pairs as reported for strain MNZ41, GenBank accession # ASJQ00000000.1. The efficiency of this reaction was 107, and the limit of detection was 42.8 GEq. These reactions were run as previously mentioned using PerfeCTa SYBR Green SuperMix Kit (Quanta Bio), a CFX96 Real-Time PCR Detection System (Bio-Rad), and 400 nm of each primer (F_RT_lytFN1 and R_RT_lytFN1). The GEq of *lytFN1*, and therefore mRNA copies, was calculated using the Bio-Rad CFX manager software.

### Cell culture

Human promyelocytic leukemia cell line HL-60 (CCL-240) was obtained from the American Type Culture Collection (ATCC). HL-60 cells were maintained at 1×10^6^ cells/mL in Iscove’s Modified Dulbecco’s Medium (IMDM) (Gibco) supplemented with 20% heat-inactivated fetal bovine serum (FBS), 100 U/mL penicillin, and 1 mg/mL streptomycin and cultured in an incubator set at 37°C with 5% CO_2_. Cells were routinely counted to maintain a low population density. The cells were differentiated into neutrophils using routine media supplemented with 1.5% dimethyl sulfoxide (DMSO) that had been filter sterilized for a period of five to seven days. Prior to use in the assay, the cells were washed in IMDM media and viable cells were counted based on trypan blue exclusion using a hemocytometer. HL-60 cells were then suspended in IMDM + 1% bovine serum albumin (BSA) at a concentration of 2×10^6^ cells/mL for use in the modified surface killing assay.

### Modified surface killing assay

Bacterial stocks were thawed and diluted to a concentration of 5×10^3^ CFU/mL in 1X PBS. After dilution, 10 *μ*L of the bacteria was plated on the surface of blood agar plates (BA) in six spots. The spots were allowed to soak into the plate by air drying at room temperature. After the spots were dry, 20 *μ*L of the 2×10^6^ HL-60 cells/mL were plated directly over three of the six spots to completely cover each spot. The spots with the freshly added HL-60 cells were also allowed to dry via air drying at room temperature. The BA plates were incubated overnight at 37°C with 5% CO_2_. Following overnight incubation, the CFUs (~30–50 colonies) in each spot were counted, and percent killing was calculated.

### H_2_O_2_ resistance assay

Bacterial stocks were thawed and diluted in THY to a concentration of 1×10^7^ CFU/mL in a 96-well plate. Samples were treated with either 2.5 mM H_2_O_2_ in triplicate or mock treated. Samples were incubated for 2 hours at 37°C with 5% CO_2_. After treatment, samples were serially diluted and plated on BA in duplicate. The data represent at least three independent experiments.

### Statistical analysis

Survival data were analyzed by the Mantel-Cox log rank test using Prism 10 software (GraphPad Software version 10 (released 2023), Inc., San Diego, CA). Phagocytosis survival and H_2_O_2_ resistance experiments were analyzed by unpaired *t* tests with 95% confidence intervals using Prism 10 software. Bacterial burden in murine tissues were analyzed using a nonparametric Mann-Whitney *U* test (two-tailed). A *P* value of <0.05 was considered to be statistically significant.

## Data Availability

Publicly available datasets were analyzed in this study. This data can be found here: https://journals.asm.org/doi/suppl/10.1128/mbio.02097-17/suppl_file/mbo001183686sd1.xlsx and https://static-content.springer.com/esm/art%3A10.1186%2Fs12866-018-1167-y/MediaObjects/12866_2018_1167_MOESM2_ESM.pdf.
