# Supplementary material for: AliC and AliD of nonencapsulated Streptococcus pneumoniae enhance virulence in a Galleria mellonella model of infection by contributing to reactive oxygen species resistance
Source: Front Cell Infect Microbiol. 2025 Jun 11;15:1583375. doi: 10.3389/fcimb.2025.1583375 (PMC12187840; doi:10.3389/fcimb.2025.1583375)
Supplement: Supplementary file 1 [file DataSheet1.pdf]

SUPPLEMENTARY MATERIAL

MATERIALS/METHODS

Growth Curves. Pneumococcal strains were grown to mid-log phase and diluted in a 96-well plate containing THY at a ratio of 1:100 for growth curve analysis. Growth curves were performed using a Stratus plate reader (Cerillo, Charlottesville, VA). Cells were grown at 37℃ with 5% CO_2 and OD_{600} measured every 10 minutes for 16 hours. Area under the curve (AUC) analysis was performed for growth within the first 1000 minutes of each replicate. AUC values were obtained using TRAPZ analysis with R package DescTools. A minimum of three independent experiments were performed with each replicate grown in triplicate.

FIGURE LEGENDS

Supplementary Figure 1. Kaplan-Meier Analysis of Survival in the *G. mellonella* Model Infected with Wildtype NESp and Respective Mutants. *G. mellonella* larvae were infected with 5×10^8 CFU/mL of each pneumococcal strain, and survival was monitored for 72 hours. Kaplan-Meier survival curves were determined for WT NESp (MNZ41) and isogenic mutants JLB02 (MNZ41Δ*aliC*), JLB04 (MNZ41Δ*aliD*), CDT01 (MNZ41Δ*hpf*), CDT02 (MNZ41Δ*SP6UMMC_07241*), CDT03 (MNZ41Δ*msbA*), CDT06 (MNZ41Δ*malX*), and CDT07 (MNZ41Δ*ytrB*). Data from at least three biological replicates, n=10 larvae per replicate, were used to calculate median values for graphs. Survival curves were analyzed for statistical significance using the log-rank test. A *P* value of <0.05 was considered to be statistically significant.

Supplementary Figure 2. Growth Curves of WT Pneumococci and Respective Mutants Cultured in THY Broth. Growth curve analysis was performed for strains MNZ41 (WT NESp), JLB01 (MNZ41Δ*aliC*Δ*aliD*), CDT04 (MNZ41Δ*lytFN1*), or CDT05 (MNZ41Δ*mgtC*) (A), for strains SPJV40 (WT encapsulated serotype 38) and CDT11 (SPJV40Δ*aliD*) (B), and for strains R36A (WT unencapsulated laboratory strain) and CDT08 (R36A;pABG5::*aliD*) (C). Growth curve analysis was also performed for NESp MNZ41 isogenic mutant strains JLB02 (MNZ41Δ*aliC*), JLB04 (MNZ41Δ*aliD*), CDT01 (MNZ41Δ*hpf*), CDT02 (MNZ41Δ*SP6UMMC_07241*), CDT03 (MNZ41Δ*msbA*), CDT06 (MNZ41Δ*malX*), and CDT07 (MNZ41Δ*ytrB*) (D). There was no significant growth defect between any of the strains tested, but a significant increase in growth of JLB01 compared to MNZ41 was observed (*P*=0.0211). Data is representative of three independent studies performed in triplicate. Bar graph is representative of results from three independent experiments performed in triplicate. Error bars represent the standard errors of the means.

Supplementary Figure 3. Modified Surface Killing Assay of WT NESp and Respective Mutants. Pneumococcal strains were exposed to differentiated HL-60 cells at a ratio of 1:100, and phagocytosis survival percentages were calculated. The percentages of survival after phagocytosis of strains MNZ41 (WT NESp), JLB02 (MNZ41Δ*aliC*), JLB04 (MNZ41Δ*aliD*), CDT01 (MNZ41Δ*hpf*), CDT02 (MNZ41Δ*SP6UMMC_07241*), CDT03 (MNZ41Δ*msbA*), CDT06 (MNZ41Δ*malX*), and CDT07 (MNZ41Δ*ytrB*) were calculated by comparing the CFU of strains

incubated with and those incubated without neutrophils. Bar graph is representative of results from three independent experiments performed in triplicate. Error bars represent the standard errors of the means.

Supplementary Figure 4. H_2O_2 Resistance of WT NESp and Respective Mutants. Pneumococcal strains were treated with 2.5 mM of H_2O_2 in THY or with THY only for 2 hours, and serial dilutions were plated on BA. Resistance to H_2O_2 was calculated for NESp strains MZN41 (WT), JLB02 (MNZ41Δ*aliC*), JLB04 (MNZ41Δ*aliD*), CDT01 (MNZ41Δ*hpf*), CDT02 (MNZ41Δ*SP6UMMC_07241*), CDT06 (MNZ41Δ*malX*), and CDT07 (MNZ41Δ*ytrB*) by comparing the CFU of strains incubated with and those without H_2O_2. Experiments were performed in triplicate, and the data are shown as the mean of triplicate wells. A *P* value of <0.05 was considered to be statistically significant.

Supplementary Figure 1

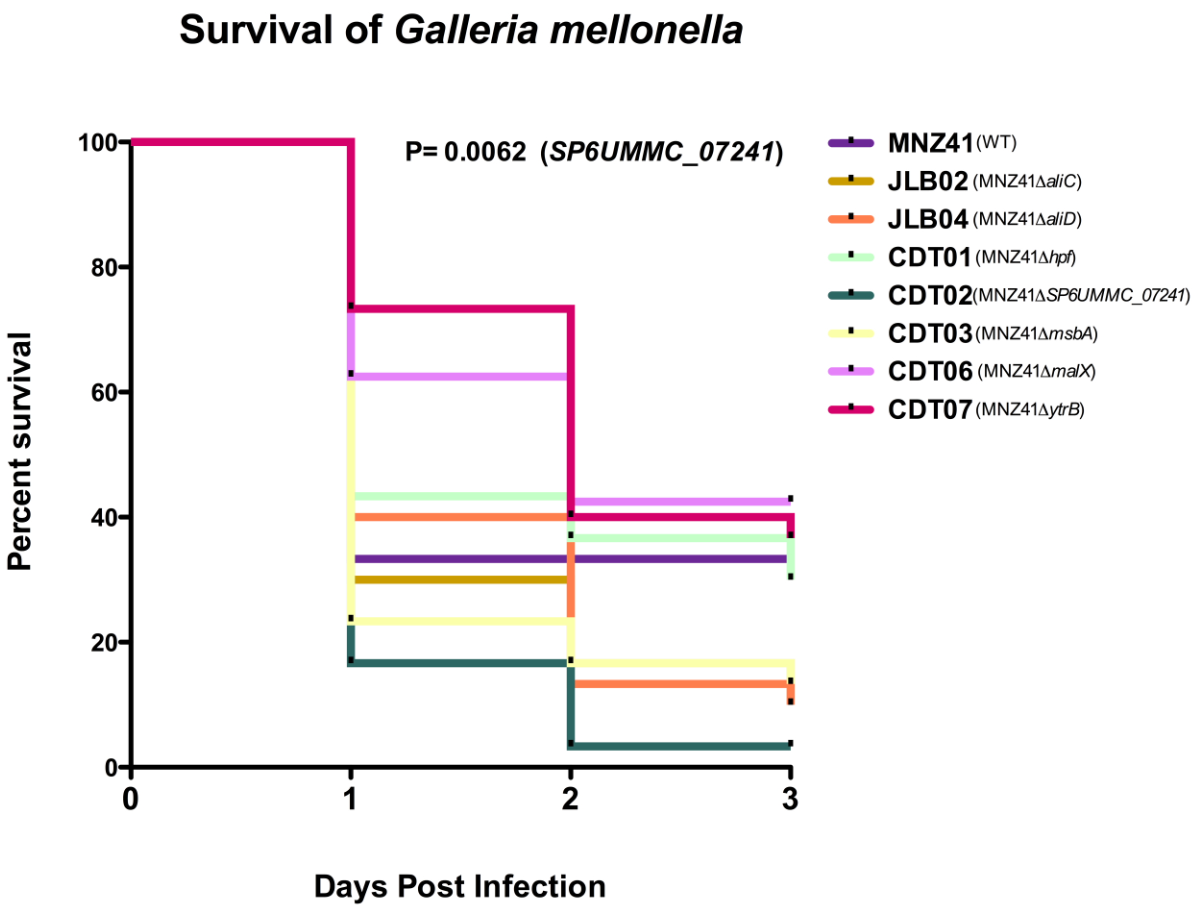

Supplementary Figure 2

Growth Curve Analysis With Area Under Curve

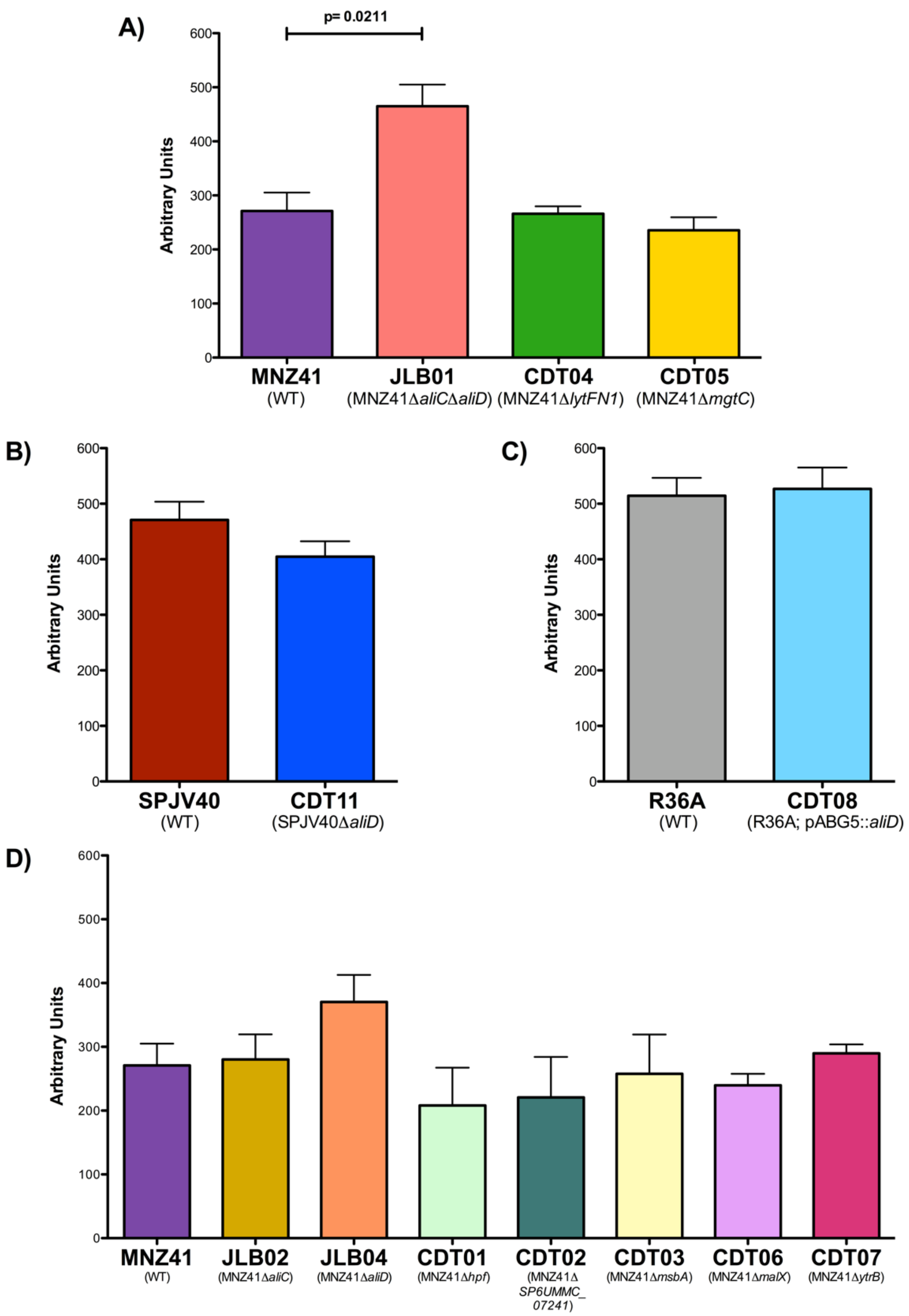

Supplementary Figure 3

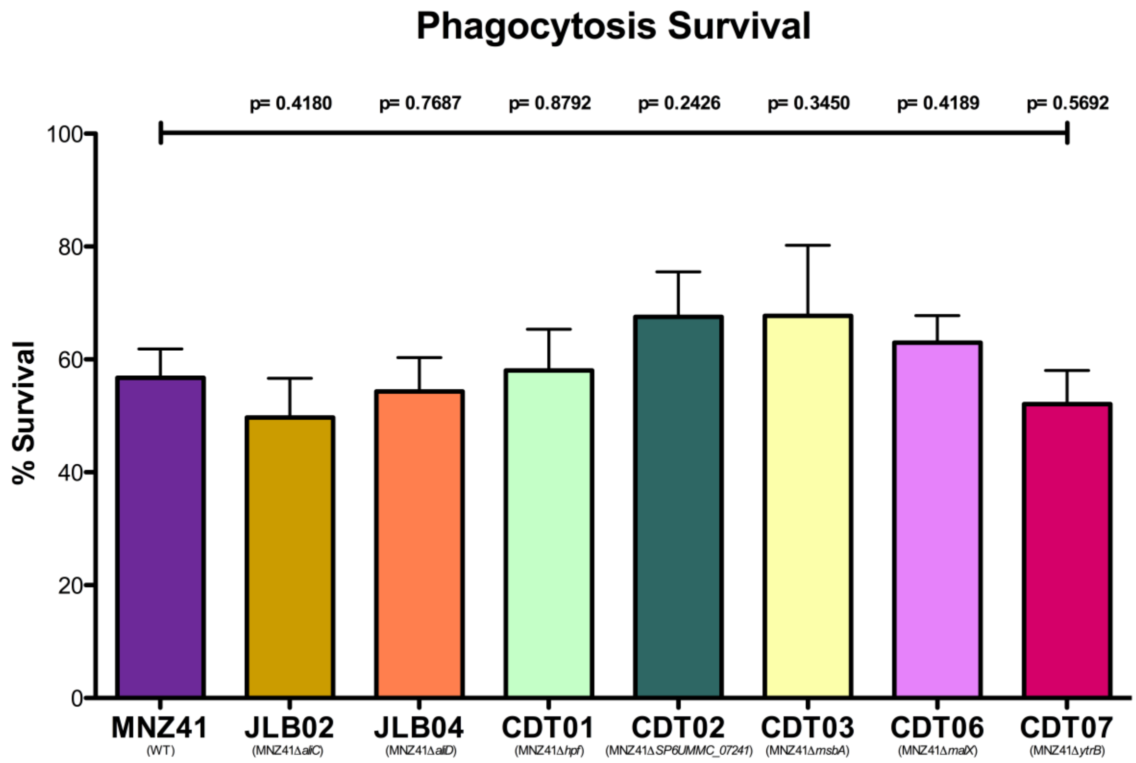

Supplementary Figure 4

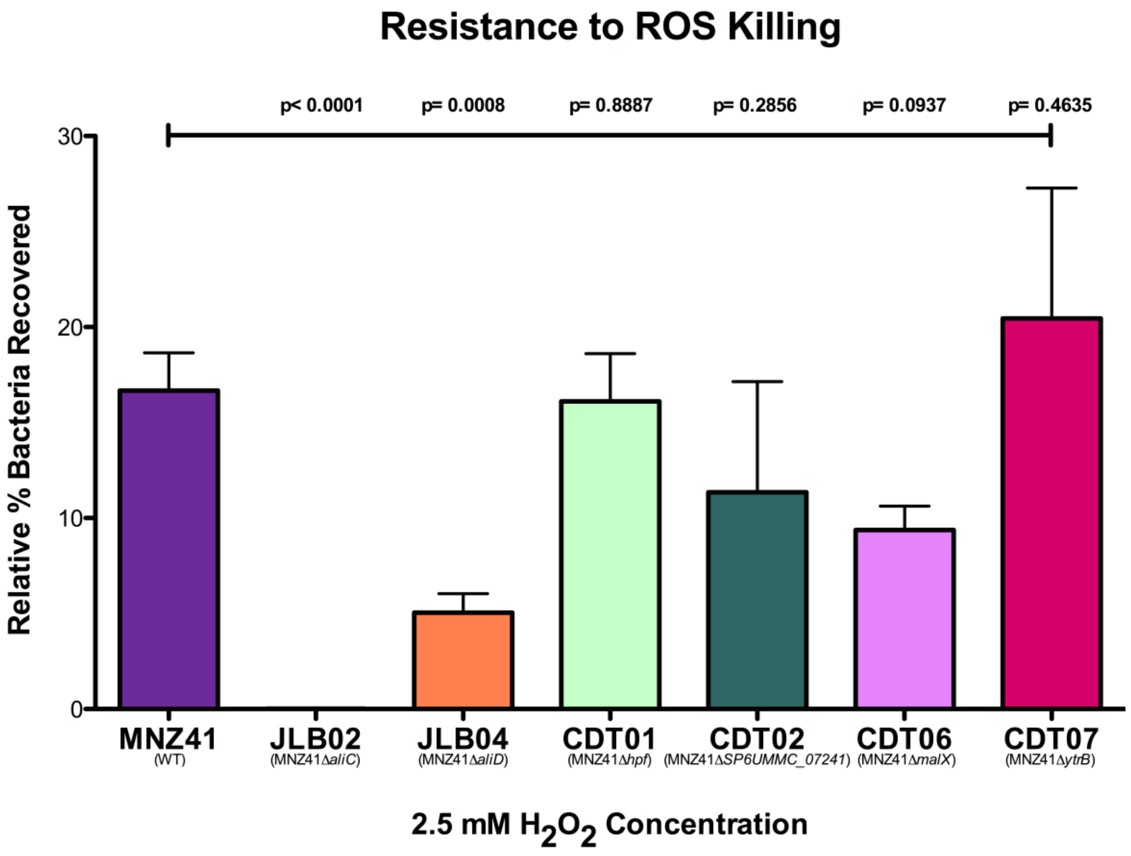

Supplementary Table 1.

Supplementary Table 1. Primer sequences used to make CDT knockout strains

Primer Name	Primer Sequence
lytFN1_Up_F	TTA TTT GTC TAA TTT TTT AGG AAT TAT ATC TCT
lytFN1_Up_R	CCA **GGT CTC** CAG GTG ATA TTA TTG TCC TTT CTA TTT TAT TTT TG
lytFN1_Down_F	CCA **GGT CTC** CGC ATG GGG GCA ATA AAA TGA AAG
lytFN1_Down_R	AGT TCT TTA GAT AAT ACC AAG AGC
mgtC_Up_F	TAA ATA CTG AAT TCT GAT ATC ATC TAC A
mgtC_Up_R	CCA **GGT CTC** CAG GTA ATT TAG TAA ACA AAG GGA C
mgtC_Down_F	CCA **GGT CTC** CGC ATG ATT ATT TCC TTA CTT TAC G
mgtC_Down_R	CAA TGA TTA TTA CAT TTG CAG GG
Hpf_Up_F	GTA CTT CAG CCA AGT TCA AA
Hpf_Up_R	CCA **GGT CTC** CAG GTT TTC AAT ATG TGT AAA GGT AG
Hpf_Down_F	CCA **GGT CTC** CGC ATA TGA GTA CCT TCT TTC TAA AC
Hpf_Down_R	TTT TCG AGA TGG AGA ACT GA
7241_Up_F	TCT ATG TAG GAA CAG AAG AAG C
7241_Up_R	CCA **GGT CTC** CAG GTA GGT AAC TCC TTT TAT AAA TAG
7241_Down_F	CCA **GGT CTC** CGC ATG AAC TAT CGG ATT AAG GAA
7241_Down_R	ATT TTG ATA ACA GAT ATT CAT AGA TAT CTC
MsbA_Up_F	GAA AAA TGA AGC GGT GTC A
MsbA_Up_R	CCA **GGT CTC** CAG GTG GAA AAT CGG TAT CAG GAC A
MsbA_Down_F	CCA **GGT CTC** CGC ATA AAT ACT CCT TAT AAT ATT TC
MsbA_Down_R	AAA AGA CTT TGG TGA AGT TGT
MalX_Up_F	ATT TTG AGC TGG TGC CCA
MalX_Up_R	CCA **CGT CTC** CAG GTG TAC TTA CTG ACT TAA TAA AAA AC
MalX_Down_F	CCA **CGT CTC** CGC ATA AAA CTC CCC CTC ACA TC
MalX_Down_R	TTT GAC AGG TGA AGC TGC
YtrB_Up_F	AGC CAC GTT TTC GAT GTC
YtrB_Up_R	CCA **CGT CTC** CAG GTA GTA TCA TAA AAG TCA GAG AG
YtrB_Down_F	CCA **CGT CTC** CGC ATC GTT TAT CCT TTC TTA AAT TTC
YtrB_Down_R	TCT CCT CTT GGA TGG AAT TC
Kan_BsaI_F	CCA **GGT CTC** CAC CTC TGC TCG AAG ATT TCA GCT TGA C
Kan_BsaI_R	CCA **GGT CTC** CAT GCC AGT GTG GTC GTA CAT CTT TGT AAC
Kan_BsmBI_F	CCA **CGT CTC** CAC CTC TGC TCG AAG ATT TCA GCT TGA C
Kan_BsmBI_R	CCA **CGT CTC** CAT GCC AGT GTG GTC GTA CAT CTT TGT AAC
AliD_EcoRI_pABG5_F	CC**G AAT TC**G AGA GTT CTT AGT GCA GG
AliD_PstI_pABG5_R	CC**G ACG TC**C TAT TCT TCT TTT TGG GC

Supplementary Table 2.

Supplementary Table 2. Primer sequences used for RT-qPCR Experiments

Primer Name	Primer Sequence
F_RT_lytFN1	CGA AGG CGA TGT GAA CTA TCT
R_RT_lytFN1	GTT CCA GCA TGA CAA CAA TCC
F_RT_mgtC	GGT TTA GAG AGA GGG AGC AAA TC
R_RT_mgtC	ACC TGA TAT AAC TTG AGC TCC TAA TC
F_RT_gyrA	CCC ATA GTT GCA CGT CCT GT
R_RT_gyrA	TCG TGG TGG TAA GGG AAT GC
